# Quantitatively Unraveling Hierarchy of Factors Impacting Virgin Olive Oil Phenolic Profile and Oxidative Stability

**DOI:** 10.3390/antiox11030594

**Published:** 2022-03-20

**Authors:** Maja Jukić Špika, Zlatko Liber, Cinzia Montemurro, Monica Marilena Miazzi, Ivica Ljubenkov, Barbara Soldo, Mirella Žanetić, Elda Vitanović, Olivera Politeo, Dubravka Škevin

**Affiliations:** 1 Institute for Adriatic Crops and Karst Reclamation, Put Duilova 11, 21000 Split, Croatia; mirella.zanetic@krs.hr (M.Ž.); elda.vitanovic@krs.hr (E.V.); 2 Centre of Excellence for Biodiversity and Molecular Plant Breeding (CoE CroP-BioDiv), Svetošimunska Cesta 25, 10000 Zagreb, Croatia; zlatko.liber@biol.pmf.hr; 3 Department of Biology, Faculty of Science, University of Zagreb, Marulićev Trg 9a, 10000 Zagreb, Croatia; 4 Department of Soil, Plant and Food Sciences (DiSSPA), University of Bari Aldo Moro, 70126 Bari, Italy; cinzia.montemurro@uniba.it (C.M.); monicamarilena.miazzi@uniba.it (M.M.M.); 5 Spin Off Sinagri s.r.l., University of Bari Aldo Moro, 70125 Bari, Italy; 6 Support Unit Bari, Institute for Sustainable Plant Protection, National Research Council of Italy (CNR), 70125 Bari, Italy; 7 Faculty of Science, University of Split, Ruđera Boškovića 33, 21000 Split, Croatia; ivica.ljubenkov@pmfst.hr (I.L.); barbara@pmfst.hr (B.S.); 8 Faculty of Chemical Technology, University of Split, Ruđera Boškovića 35, 21000 Split, Croatia; olivera@ktf-split.hr; 9 Faculty of Food Technology and Biotechnology, University of Zagreb, Pierottijeva 6, 10000 Zagreb, Croatia; dskevin@pbf.hr

**Keywords:** phenolic profile, secoiridoids, *Olea europaea* L., environmental conditions, principal component analysis, molecular identification, DNA, traceability, cv. Oblica, cv. Leccino

## Abstract

A single phenolic group and even a compound play different roles in the sensory properties and stability of virgin olive oil (VOO), which in turn are strongly influenced by several factors. Understanding the causes of differences in phenolic compound composition and oxidative stability (OS) in VOOs is essential for targeted and timely harvest and processing while maintaining desired oil quality. The phenolic profile and OS of two monocultivar VOOs (Oblica and Leccino) grown in two geographical sites of different altitudes (coastal plain and hilly hinterland) were analyzed throughout the ripening period over two years. Concentration of secoiridoids was 30% higher in the Oblica than in the Leccino VOOs, which in turn had significantly higher values of OS. Both cultivars had more than twice as high concentrations of the two most abundant phenolic compounds, the dialdehyde form of decarboxymethyl oleuropein aglycone and the dialdehyde form of decarboxymethyl ligstroside aglycone, and OS values in a colder growing site of higher altitude. Among the studied monocultivar VOOs, the secoiridoid group did not behave equally during ripening. The hierarchy of different influencing factors was investigated using multivariate statistics and revealed: cultivar > geographical site > harvest period > growing season. In addition, the possibility of traceability of VOO using molecular markers was investigated by establishing SSR profiles of oils of the studied cultivars and comparing them with SSR profiles of leaves.

## 1. Introduction

Integrating comprehensive profiling approaches with multivariate statistics allows us to access complex biological systems, such as the olive. The natural fruit juice of the olive tree (*Olea europaea* L.) is virgin olive oil (VOO) and is the main source of fat for populations adhering to the Mediterranean diet worldwide, especially in Mediterranean countries. 

Extra virgin olive oil stands out as a source of antioxidants that contribute to a balanced diet, which is very important nowadays because the opposite leads to an imbalance between the formation of free radicals and the ability to remove radicals that cause oxidative stress [1,2]. The oxidative stress induced by free radicals is the cause of many diseases, mainly through the induction of reactive oxygen species (ROS), which in turn can cause DNA damage [2,3,4]. Phenolic compounds from VOO have been shown to have potent antioxidant activity that can directly scavenge some radical species and minimize the amount ROS generated by fatty acid peroxidation [3,5]. Thus, this naturally occurring antioxidants from sources such as VOO have become a cost-effective alternative for oxidative stress prevention. 

A large number of phenolic compounds belonging to several groups have been isolated and identified in VOO: flavonoids (apigenin, luteolin), phenolic alcohols (tyrosol, hydroxytyrosol), phenolic acids (caffeic acid, vanillic acid), and a predominant group of secoiridoids that account for 60–90% of total phenolics [6,7]. A single group of phenols and even one phenolic compound play a different role in sensory properties [8] and oil stability [9,10]. The concentration of phenolic compounds is strongly influenced by numerous factors, from genetic [11] and agronomic [12,13] to various technological aspects [14,15,16,17]. In the last decade, at the top of mentioned, attention has been given to the studies on the adaptation of autochthonous and allochthonous cultivars to edaphoclimatic conditions’ changes [18,19] and the selection of new olive cultivars [20] with a focus on phenolics. All of this due to the expansion of olive cultivation to arid olive areas, as well as climate changes that could lead to changes in the properties of oils obtained from olives grown in their original habitat. The phenolic compounds in the olive fruit change in direct relation to temperature, rainfall (amount of water absorbed), and the nature of the soil, so that the resulting oil has a very different phenolic profile, leading to different conclusions. Monitoring changes is even more complicated when an additional factor is added, such as latitude and/or altitude of the olive grove. Consequently, the literature shows a large variation between and within cultivars in the levels of phenolic compounds in VOOs [21,22,23,24,25,26]. They respond differently to the influence of the same factors. To some extent, this is to be expected, as geographical location, harvest period and other previously mentioned elements determine relationships with both, olive fruit constituents (phenolic compound precursors) and the activity/capacity of enzymes that form the final picture of phenolic concentration in VOO. Thus, this requires significantly more research and knowledge of different monocultivar VOOs obtained from different growing areas and under different environmental conditions to answer how and why individual monocultivar oils and/or all of them react.

The aim of this study was to determine the changes in phenolic profile and oxidative stability of VOOs of the allochthonous Italian cultivar Leccino and the autochthonous Dalmatian cultivar Oblica at different fruit ripening stages, both grown in two different growing sites (flat coastal plain and hilly hinterland). Furthermore, we investigated the influence of environmental conditions (temperature and rainfall) recorded during two growing seasons on the studied VOO properties and we determined dependencies and statistical significance of VOO components with the oxidative stability. The focus of the study was on the determination of the main sources of variation within the results caused by the observed four factors (cultivar, growing season, growing site and harvest period) using multivariate statistics. It should be emphasized that a substantial contribution to the knowledge of monocultivar VOOs from a particular terroir allows achieving a higher level of oil quality. The integrity of such VOOs is at high risk, as they are protected by labels such as Protected Designations of Origin or Protected Geographical Indications, which allows the producer to obtain a higher income. These oils, whose quality is strictly dependent on the cultivar used and the territory in which it is grown, can be adulterated with inferior (lower quality) and cheaper oils [27]. To preserve the integrity of such oils, DNA profiling technologies are becoming increasingly important as they are rapid, reliable, and objective methods that directly compare genetically inherited material [28,29]. Therefore, the aim of this study was also to evaluate the possibility of olive oil traceability using molecular markers by generating SSR profiles of oils of studied cultivars and comparing them with leaf SSR profiles.

## 2. Materials and Methods

### 2.1. Olive Samples and Oil Extraction

The samples of olive fruit from the autochthonous Croatian cultivar Oblica and the allochthonous Italian cultivar Leccino were harvested from two olive orchards in two consecutive years (2011 and 2012). The orchards are located in two very different olive sub-regions: in the flat coastal plain site Kaštela (43°55′ N; 16°35′ E, 28 m above sea level) and in the hilly hinterland site Šestanovac (43°27′ N; 16°55′ E, 358 m above sea level). The climate types are defined as Csa and Cfa, respectively [30]. The characteristics of the olive groves and the climatic parameters prevailing in the years studied were described in detail in the previous study [31]. In brief, the average daily temperatures and precipitation recorded at growing sites (Table S1) show that Šestanovac is associated with more rainfall and lower temperatures, which characterizes Šestanovac as a less drought-affected and colder growing site compared to Kaštela.

In each growing season, healthy olives were sampled from all sides of the canopy of each of the three sampled trees that correspond to one lot. Lots were produced from green to ripe olives over 4 harvest periods (HP) beginning in late September with a 14-day interval between harvests. Maturity index (MI) was determined using a representative subsample (100 fruits) from each homogenized olive lot [32]. The maturity index ranged from 0.0 to 3.94 for Oblica fruits and from 1.05 to 4.10 for Leccino (Table S2) [31]. In general, MI of Leccino was higher compared to Oblica fruits at both growing sites during the observed ripening period. Oblica fruits colored almost uniformly at both sites, while Leccino fruits from Šestanovac ripened about 15 days slower compared to those from Kaštela, with MI increasing more at the end of the studied ripening period. 

Olive oil was extracted within 24 h from harvest by centrifugal extraction in a laboratory oil mill (Abencor, MC2 Ingenieria y Sistemas, Seville, Spain) that simulates the industrial process of VOO production. After grinding the olives, the olive paste was kneaded for 35 min at 26 ± 2 °C. The oil was collected by vertical centrifugation at 1370× *g* for 70 s and decanted. The oil mill was washed between each batch of olive fruit. After centrifugation and decantation, the obtained oil samples were stored in glass bottles and in the dark at 16–18 °C. According to the protocol established in the EU regulations [33], all oil samples were determined as extra virgin olive oil. All analyses were performed in triplicate.

### 2.2. Analyses of Phenolic Compounds

#### 2.2.1. Extraction of Phenolic Compounds 

Extracts for phenolic profile determination were prepared according to the modified procedure of Gutfinger [34]. Internal standard (syringic acid at the concentration of 0.015 mg mL^−1^) was added to the olive oil sample, and the mixture was shaken for 30 s. Liquid–liquid extraction of a mixture in *n*-hexane with a water/methanol mixture (60:40, *w*/*w*) was performed three times. The combined hydroalcoholic phases were evaporated on a rotary evaporator at 40 °C (Devarot, Elektromedicina, Ljubljana, Slovenia). The dry extracts were then dissolved in methanol and filtered through a 0.45-µm polyvinylidene difluoride filter (Whatman, Buckinghamshire, UK).

#### 2.2.2. Identification and Quantification of the Individual Phenolic Compounds

The phenolic compounds of the extracts were separated using the Perkin Elmer high-performance liquid chromatography HPLC system (Waltham, MA, USA) equipped with a variable UV/VIS detector at 280 nm and the TotalChrom Workstation software package. Separation of phenolic compounds was achieved on a C18 column (Ultra-Aqueous C-18, 250 × 4.6 mm, 5A) (Restek, Bellefonte, PA, USA) by gradient chromatography. The flow rate was 0.8 mL/min. The mobile phase used consisted of 0.2% phosphoric acid (A), methanol (B), and acetonitrile (C) for a total run time of 80 min. The initial conditions were 96% A, 2% B, and 2% C. During 40 min, the ratios were changed to 50% A, 25% B, and 25% C, and from 40 to 45 min, the ratios were changed to 40% A, 30% B, and 30% C. From 45 to 60 min, the gradient was 50% B and 50% C, held until 70 min, and then returned to the initial conditions over 10 min. Identification was made by comparing retention times with those of the pure standard (hydroxytyrosol, tyrosol, oleuropein, vanillin, caffeic acid, syringic acid, *p*-coumaric acid, ferulic acid, luteolin, and apigenin) or by comparing retention times and absorbance [35]. Quantification of phenolic compounds (previously mentioned and identified by standard substances) was performed using the calibration curve of the standard, and results were expressed in mg of each phenolic compound per kg of olive oil. For other compounds, quantification was based on the internal standard (calculation of the relative response factor between syringic acid and tyrosol) and results were expressed as tyrosol. The standard and solvents were of analytical grade and were purchased from Sigma-Aldrich (Steineheim, Germany). Deionized water (Milli-Q, Millipore, Bedford, MA, USA) was used for the preparation of all solvents.

### 2.3. Oxidative Stability Analysis

Determination of the oxidative stability (OS) of VOOs was performed in a Rancimat Metrohm 743 instrument (Metrohm, Herisau, Switzerland). The sample (2.5 g of VOO) was placed in the reaction tubes of the electrically heated block and subjected to thermal degradation at 110 °C by constantly blowing a stream of air into the reaction tube at a rate of 20 L h^−1^. The air containing volatile organic acids from the oil sample was collected in a dosing vessel containing 60 mL of deionized water. Continuously, the water conductivity was recorded and the induction time, as the OS of the oil at a given temperature, was determined automatically. The results are expressed in hours, i.e., the time period during which the oil resisted to oxidative stress.

### 2.4. Statistical Analysis 

All data were statistically analyzed using Statistica 14.0.0.15 (Tibco Software Inc, Palo Alto, CA, USA, 2020). Descriptive statistics were generated for the entire data set from two cultivars in two consecutive years, two geographical sites, and from four harvest periods. Cultivars differed significantly in five of the 16 traits examined (factorial analysis of variance (ANOVA)), so an independent analysis was performed for each cultivar. Data from each cultivar were used to evaluate the relative contribution of growing season, growing site, and harvest period on the variability of phenolic and oxidative stability in VOO samples using a three-way analysis. Separation of means was achieved at *p* ≤ 0.05 by Tukey’s honestly significant difference test. 

Pearson’s linear correlation matrix (with *p* ≤ 0.05 as statistically significant) was used in order to determine the extent to which phenols or a group of phenols and recorded oxidative stability were related to the climatic conditions that prevailed during the year. The correlation coefficients were also used to determine the statistical relationship between VOO constituents and their oxidative stability. Regarding the constituents of VOO, in addition to the data presented in this paper work (composition of phenolic compounds), data on tocopherol content (α-, γ-tocopherol and total tocopherols) and fatty acid composition presented in Jukić Špika et al. [31,36] were also used. 

Principal component analysis (PCA) was used to quantify the degree of association between phenolic compound composition and oxidative stability with the four factors studied. PCA was applied to the entire data set (two genotypes, two growing seasons, two growing sites, and four harvest periods).

### 2.5. Genetic Identification 

Leaf tissue of Oblica and Leccino cultivars was sampled in 2014 to evaluate the possibility of olive oil traceability by comparing the simple sequence repeats (SSR) profile of the leaves with the SSR profiles of the oils. In the same year, the samples of olive oils were obtained from the fruits harvested from the tree of a certain cultivar and processed by centrifugal extraction under the same conditions as previously described. 

#### 2.5.1. DNA Extraction

DNA was extracted from both leaf tissue and extracted monocultivar oils according to Spadoni et al. [37], starting from 30 mg of freeze-dried leaves and 250 mL of oils centrifuged at 10,000 rpm for 5 min. DNA was tested for quality and quantity using both 0.8% agarose gel electrophoresis and a spectrophotometer (Nanodrop 1000, Thermo Scientific, Waltham, MA, USA). 

#### 2.5.2. SSR Amplification, Capillary Electrophoresis, and Data Analysis

Ten microsatellite markers were used for the analysis [38,39,40]. The PCR mix contained 50 ng of genomic DNA, 0.25 µM of each primer, 0.2 mM each dNTP, 2 mM MgCl_2_, 1 × Euroclone reaction buffer and 2 U EuroTaq DNA polymerase (Euroclone^®^, Milan, Italy) in a total volume of 25 µL. The forward primer was labeled with FAM and HEX fluorochromes (Sigma-Aldrich, St. Louis, MO, USA). PCR reactions were performed in a C1000™ Thermal Cycler (Bio-rad, Hercules, CA, USA) under the following conditions: 94 °C for 5 min, 35 cycles at 94 °C for 30 s, 50 to 60 °C (depending on the SSR primer combination) for 30 s, and 72 °C for 30 s, and final elongation at 72 °C for 60 min. PCR products were separated using the ABI PRISM 3100 Avant Genetic Analyzer (Life Technologies, Carlsbad, CA, USA), using a mixture of 2 µL PCR reaction, 12 µL Hi-Di™ formamide (Life Technologies, Carlsbad, CA, USA), and 0.3 µL GeneScan™ 500 ROX™ size standard (Life Technologies, Carlsbad, CA, USA). Allele size was determined using GeneMapper^®^ software version 3.7 (Applied Biosystems, Foster City, CA, USA).

## 3. Results and Discussion

### 3.1. Phenolic Profile as a Function of Cultivar, Environmental Conditions, and Harvest Period 

Phenolic alcohols and secoiridoids are considered to be the main phenolic group of compounds, while phenolic acids, flavonoids, and lignans have also been identified in VOOs playing various significant and synergistic roles [7,41,42]. Table 1 and Table 2 show the phenolic composition of the studied monocultivar VOOs, which differed significantly among the examined cultivars (except for tyrosol, vanillic acid, dialdehyde form of decarboxymethyl ligstroside aglycone, apigenin). The dialdehyde form of decarboxymethyloleuropein aglycone (DMOdA) (Oblica 78.8–277.2 mg kg^−1^; Leccino 60.6–347.9 mg kg^−1^) and the dialdehyde form of decarboxymethyl ligstroside aglycone (DMLdA) (Oblica 85.1–166.5 mg kg^−1^; Leccino 32.9–142.7 mg kg^−1^) were the most abundant phenolic compounds in both monocultivar oils (Table 1 and Table 2), consistent with previously published studies for other VOOs [20,43]. The average content of hydroxytyrosol in Oblica oils was 6.2 mg kg^−1^ being significantly higher than in Leccino VOOs (average 4.0 mg kg^−1^). For lignans, a significant higher pinoresinol concentration was found in Oblica compared to Leccino VOOs, which could be a cultivar characteristic according to Brenes et al. [44].

The evaluation of the phenolic composition of VOO from two growing seasons and different geographical sites (Kaštela and Šestonovac) revealed significant differences (*p* ≤ 0.05) between seasons and sites. Differences in phenolic compound concentrations between VOO from two growing seasons were less pronounced in Oblica (Table 1) than in Leccino VOOs (concentrations were up to two times higher) (Table 2). Some researchers point out that growing season is a factor that leads to a difference in phenolic content in other monocultivar VOOs [45,46]. This is most likely due to the different climatic conditions that prevail in a given year. Accordingly, using correlations useful for indicating a predictive relationship that can be applied in practice, a significant relationship between precipitation and mean daily temperature (Table S1) was found with the concentration of phenolic compounds (Table 1, Table 2 and Table 3).

During the period of intensive olive fruit growth precipitation correlated positively with the concentration of phenolic alcohols, flavonoids (luteolin) and secoiridoids (OAgl-A, OA-dA), while it correlated negatively with phenolic acids (Table 3). The negative correlation of precipitation with seven phenolic compounds was observed in the period of fruit ripening with the strongest correlation with total secoiridoids content (TSC). In 2011, the July–August period was wetter than the same period in 2012 (166.2 and 216.6 mm, 44.6 and 61.1 mm; 2011 and 2012 for Kaštela and Šestanovac, respectively; Table S1), while olive received more water in the ripening period in 2012 (256.0 and 207.3 mm, 317.6 and 220.7 mm; 2011 and 2012 for Kaštela and Šestanovac, respectively; Table S1). The literature suggests that water availability affects the groups of phenolic compounds to varying degrees, with the greatest changes observed in the proportions of compounds from the secoiridoids group [47]. In the present study (Table 3), phenolic acids were found to be most affected by precipitation during the observation period (the correlation was significant in 10 cases out of 20 pairs studied). However, since we know the importance of secoiridoids and this group is the most abundant; it is noteworthy to timepiece their behavior. For Oblica VOOs, TSC differed only about 2% between growing seasons and was higher in 2011 (Table 1). Larger differences (about 20%) and higher TOS values in 2012 were observed in Leccino VOOs (Table 2). Within the individual fractions of secoiridoids, the highest difference between two growing seasons studied was 9% in Oblica VOOs, while in Leccino VOOs the concentrations of DMOdA and OAgl-A differed by 50% between seasons (Table 1 and Table 2). Water availability is considered as an essential parameter for phenol synthesis [19,48,49,50] by affecting (under the stress conditions) the activity of L-phenylalanine ammonium lyase [51], which most likely represents the agronomic traits of a cultivar [52,53]. Thus, the different responses of two cultivars studied may be explained by the fact that Oblica is the autochthonous cultivar better adapted to stress than Leccino, a cultivar from Tuscany destined for intensive cultivation and requiring either deep soils or irrigation in the summer months for regular fruit development and oil synthesis. Pinoresinol was the only compound for which changes in rainfall and temperature did not lead to the change in concentration during the observed period (Table 3).

The studied monocultivar VOOs differed significantly regarding two different growing sites (Table 1 and Table 2). Among the factors under observation (growing season, growing site and time of harvest) for most of the identified phenolic compounds, the highest variability (F-statistic values) was observed by the growing site. Its strongest influence was recorded on the secoiridoid group (TOS) and their fraction. Both cultivars showed higher average content in Šestanovac, a colder and higher altitude growing site (Table 1 and Table 2). Although the content of phenolic compounds is related to the content of phenolic glucosides originally present in olive fruit and is determined geographically, it is usually strongly influenced by environmental factors [20]. We found a negative correlation of the mentioned concentration of certain phenolic compounds with temperature (Table 3), which acts as a regulatory factor for different enzymes on the pathway of phenolic synthesis, leading to the changes in final concentrations. Arslan at al. [23] reported the highest concentration of DMOdA and DMLdA for Sariulak VOO at the higher and colder growing site. However, for total phenolic compounds, it was also observed that cultivars behaved differently depending on the growing site [54,55] and opposite results were also published, where a higher content was found at a lower altitude location [56,57]. In contrast to the behavior of the compounds belonging to the secoiridoids group, in the present study, the concentration of vanillic acid and pinoresinol was higher in both cultivars at the warmer, drier, and lower altitude growing site (Table 1 and Table 2). Tura et al. [56] attributed the higher vanillic acid concentration to a higher elevation site with lower average temperatures, while Arslan et al. [23] referred to a lower elevation site with higher average daily temperatures. This suggests that the variations in phenolic compounds caused by growing area are due to the several combined factors such as altitude, soil texture, temperature, the availability of water described earlier, but also to the different soil properties of studied olive orchards (loamy clay compared to moderately carbonate soil obtained by crushing the surface layer).

The chemical and enzymatic reactions that occur during ripening are reflected in the altered phenolic profile of the VOOs. In addition to total phenols [31], the degree of ripening also affects the concentrations of individual phenolic compounds [7]. Accordingly, in this study, significant differences in the content of individual phenolic compounds were found in the monocultivar Oblica and Leccino VOOs obtained from fruits with different maturity levels that ranged from 0.0 to 3.94 for Oblica fruits and from 1.05 to 4.10 for Leccino (Table 1, Table 2 and Table S2). 

Jiménez et al. [58] reported the highest content of tyrosol and hydrotyrosol in VOOs of early harvested Picudo cultivar, and stood out that at the end of the ripening the content of hydroxytyrosol decreased by 50%. In general, our study found the same pattern of changes in simple phenols with MI incensement for both cultivars (Table 1 and Table 2). Exceptions, such as an increase in the middle stages of ripening (Hyt: Oblica, Šestanovac 2012; Tyr: Oblica Kaštela 2012; Leccino, Šestanovac 2011 and Kaštela 2012) and no influence of ripening (Tyr: Oblica, Kaštela 2012) were also observed. Same changes were reported by Gallina-Toschi et al. [59] for VOOs of the Nostrana di Brisighella cultivar.

Oleuropein and ligstroside derivatives were the main phenolic fractions in all analyzed samples (Table 1 and Table 2), as previously described in VOO of Arbequina, Cornicabra, Picual, Chetoui, Chemalali and Buža [60,61,62]. Among the different varietal VOOs studied, the group of secoiridoids does not behave the same during ripening, and the results of our research confirm that. Among the Leccino VOOs, the secoiridoids were found to peak in the mid-harvest oils (Table 1 and Table 2), which is consistent with the expected accumulation line of these compounds, as reported by Baccouri et al. [61] for the monocultivar Chetoui and Chemlali VOOs. Among the identified fractions in the group of secoiridoids, a deviation from this behavior was observed only for OA-dA, VOOs from Kaštela. For Oblica VOOs, a constant decrease in secoiridoids fractions with maturation was observed (Table 1 and Table 2). A similar decrease in secoiridoids during maturation was observed in Arbequina, Cornicabra, and Picolimon [60].

The dialdehyde form of the decarboxymethyloleuropein aglycone, as well as other fractions of the secoiridoids group, are derived from the secoiridoid glucosides present in olive fruit by enzymatic action during the processing of the fruit into oil [7]. Moreover, it is known that oleuropein is the main phenolic compound of olive fruit [63], for which Ryan et al. [64] found a constant decrease with fruits ripening of the Cucco cultivar. However, in the same study, an increase in concentration was observed in the VOOs of Manzanillo cultivar, followed by a degradation and a decrease in oleuropein concentration. The changes in the phenolic compound content of the oils due to maturation followed the changes observed in the oleuropein concentrations. Thus, the aforementioned study suggests that the study period started late to observe the growth phase when oleuropein reaches its highest concentration (Ryan et al., 1999), which is also evident in the present study for Oblica VOOs (Table 1 and Table 2).

The results of flavonoid content of Oblica and Leccino VOOs are in agreement with the results of Atrajo et al. [65], who reported that the concentrations of flavonoids in VOOs increased with olive fruit ripening. The concentration of luteolin in Oblica VOOs increased with fruit ripening, with the exception in 2012 (Kaštela), where no differences were observed during ripening (Table 1). In the case of Leccino VOOs, a decrease in concentration was observed in VOOs obtained from overripe fruits (last harvest period) (Table 2), which was also observed by Jiménez et al. [58] in Picudo VOOs. Apigenin concentration increased with olive fruit ripening in both cultivars (Table 1 and Table 2).

### 3.2. Oxidative Stability as a Function of Cultivar, Environmental Conditions and Harvest Period

**Figure 1:**
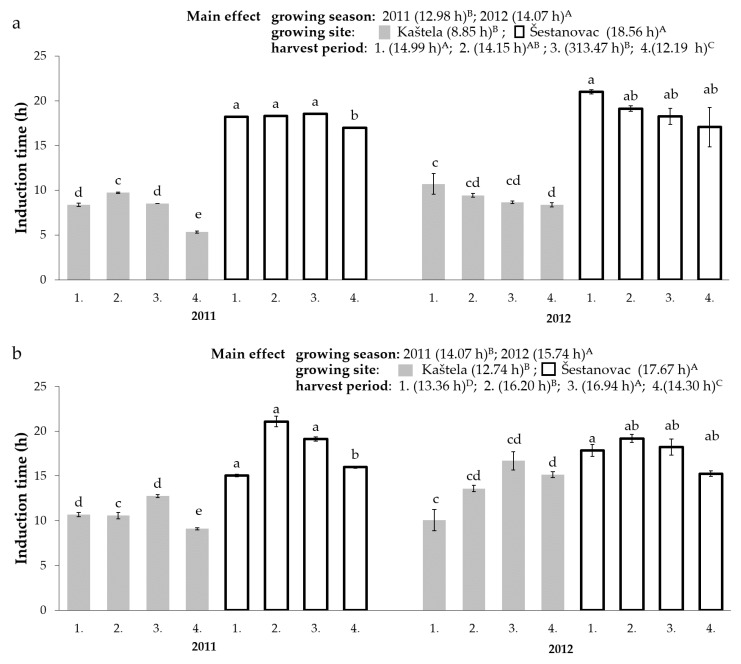
Oxidative stability of Oblica (**a**) and Leccino (**b**) virgin olive oils during ripening from two distinct olive orchards (Kaštela and Šestanovac) in two consecutive years. Bars marked with different lowercase letters for each growing season are significantly different, while different upper-case letters indicate differences within main effects (growing season, growing site, and harvest period), determined by a three-way test ANOVA (Tukey’s test, *p* ≤ 0.05). 1–4, harvest period.

The determination of the VOO oxidative stability is its viability prediction in terms of fatty acid composition and bioactive compound content. The results shown in Figure 1 indicate significant differences in the oxidative stability of Oblica and Leccino VOOs (F = 88.41, *p* < 0.001). The oxidative stability of Oblica VOOs ranged from 5.32 to 20.99 h with the average of 13.71 h for all tested samples (Figure 1). Leccino VOOs showed significantly higher oxidative stability values with an average value of 15.16 h (15.97–19.18 h) (Figure 1).

The influence of climatic conditions on VOOs oxidative stability, which differed significantly between the two observed growing seasons and growing sites, was also evaluated (Table 4). The results show that mean daily temperature during intensive olive fruits growth and ripening and rainfall in September were negatively correlated with OS. A higher average value of OS was measured in the Oblica and Leccino VOOs in 2012 (Oblica; 2011—12.99 h, 2012—14.07 h; Leccino; 2011—14.07 h, 2012—15.74 h). The reason for the lower OS values in 2011 can also be found in the loss of naturally present antioxidants (tocopherols) in specified season [36]. Deiana et al. [66] state that tocopherols act as synergists with phenolic compounds and thus have a significant antioxidant effect. Several studies have reported the tocopherol influence on the OS, highlighting the high tocopherol content of Leccino VOOs [61,67]. Moreover, a lower ratio of oleic and linoleic acids was found in the VOOs of both cultivars in 2011 [31], which is strongly correlated with oxidative stability (r = 0.71) according to Aparicio et al. [68].

Oxidative stability changes due to the harvest period are shown in Figure 1. The lowest OS was found for both cultivars in VOOs from overripe olives (4th HP). The same was reported for Cornicabra VOOs [69]. The average value of OS for Oblica VOOs (from both growing sites) (OS, Kaštela: first harvest 9.9 h, fourth harvest 7.5 h, Šestanovac: first harvest 20.0 h, fourth harvest 17.2 h) (Figure 1) decreases with MI increasing. Most probably as the result of decreasing content of phenols (Table 1), tocopherols, as well as changing fatty acid profile found for these VOOs [31,36]. For Leccino VOOs, a higher average OS value was found in the oils obtained from the second and third harvest (Figure 1), which follows the phenols changes in these monocultivar VOOs with maturation (Table 2).

### 3.3. Relationship between Oxidative Stability and Oil Constituents 

To determine the influence of fatty acid composition, tocopherol content, and phenolic composition on OS, the dependencies and statistical significance were determined using the Pearson correlation coefficient (Table 5). Correlation of OS with oleic acid and C18:1/C18:2 ratio in the positive direction (*r* = 0.653; *r* = 0.632) and the correlation of OS with linoleic acid in the negative direction (*r* = −0.593) were found. A positive correlation of low magnitude was observed between OS and α- and total tocopherol content (*r* = 0.310). Secoiridoids, among which DMOdA stood out (*r* = 0.805), showed the highest correlation with OS. The same findings were published in previous studies [59,67,70]. Nowadays, more dedicated oil producers know the cultivars in their olive groves, as well as they are at least roughly aware of the chemical data of the VOOs. Shelf life is a mandatory parameter on the product label and the producer bears the consequences if the olive oil is not properly categorized within the time frame foreseen for the market. Therefore, the results of the relationship between the oil properties and OS could be very important for the VOOs producers to evaluate and get a complete picture of the VOOs stability as an indicator of the shelf life.

The Oblica VOOs had a higher concentration of phenolic compounds compared to the Leccino VOOs (Table 1 and Table 2). On the other hand, unfavorable fatty acid composition with significantly higher linoleic fatty acid content (Oblica 11.22%, Leccino 6.99%) and lower C18:1/C18:2 ratio was characteristic for Oblica compared to Leccino VOOs (Oblica 6.46, Leccino 11.74) [31]. Alpha-tocopherol in significantly lower concentration was also detected in Oblica VOOs (Oblica 295.68 mg kg^−1^, Leccino 493.2 mg kg^−1^) [36]. Although the phenolic compounds concentration was the most strongly correlated with OS (Table 4) [21,59,68], Oblica oils were characterized with a higher C18:1/C18:2 ratio as well as lower tocopherols compared to Leccino oils [31,36], that ultimately resulted in the lower average OS of Oblica versus Leccino VOOs in this study (Figure 1).

### 3.4. Multivariate Statistics to Identify the Hierarchy of Variance of the Virgin Olive Oil Phenolic Profile and Oxidative Stability

**Figure 2:**
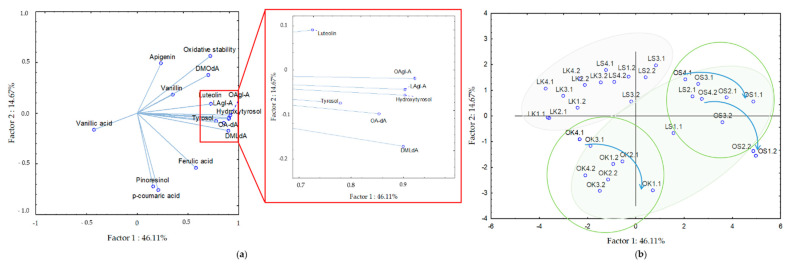
(**a**) Diagram of the loadings for the composition of phenolic compounds and oxidative stability obtained by principal component analysis; and (**b**) projection of the samples of Oblica (O) and Leccino (L) virgin olive oils grown in Kaštela (K) and Šestanovac (S) during fruit ripening (1–4, harvest period) in two growing seasons (1–2, number after the point).

Determining the main cause of variability in the composition of phenolic compounds and OS may be of great interest to olive oil producers in order to carry out targeted and timely harvesting and processing of the oil while maintaining the desired oil quality. In our previous report [31], we showed that among other studied VOO characteristics, the content of total phenolics significantly depends on the cultivar. Therefore, the focus of the present study was on the specifics of phenolic composition and determination of the hierarchy of factors affecting its concentration using multivariate statistics. We subjected the entire data set (two genotypes, two growing seasons, two growing sites, and four harvest periods) to principal component analysis. The results showed that the first five principal components had eigenvalues greater than 1 and together explained 87.21% of the variance. The first principal component explained 46.11% of the total variance. A strong positive correlation with factor 1 was found for tyrosol, hydroxytyrosol, and the group of secoiridoids, luteolin, and OS, whereas the correlation for vanillic acid was negative (Figure 2a). The second principal component explained 14.67% of the total variance and was negatively correlated with pinoresinol and *p*-coumaric acid (Figure 2a). The projection also shows the strongest positive correlation of OS with the content of DMOdA (Figure 2a), which is consistent with the research of Žanetić et al. [71], according to which secoiridoid derivatives are responsible for antioxidant activity in native Dalmatian VOOs.

The cultivar was the first grouping variable (Figure 2b). Samples from Leccino VOO were in the fourth quadrant (negatively correlated with factor 1 and positively correlated with factor 2). Virgin olive oils obtained from Leccino cultivar grown in Kaštela site were strongly negatively correlated with factor 1, where vanillic acid was isolated, while VOOs from the Šestanovac site were positively correlated with factor 2 and had low pinoresinol content. The first two principal components related to growing site separated samples of Oblica VOOs more strongly compared to Leccino VOOs. Oblica VOOs from Šestanovac were located in the first quadrant (positively correlated with factor 1 and factor 2) and were characterized by the higher content of simple phenols and secoiridoids (Figure 2a,b). On the diagonally opposite side were the Oblica VOOs from Kaštela (negatively correlated with factor 1 and factor 2). A recent study using multivariate analysis showed that olive grove altitude plays an important role in differentiating olive oil samples from a single cultivar [72].

In the present study, VOOs from the colder and wetter site of higher altitude had higher concentrations of secoiridoids and OS. In addition, the example of Oblica shows more clearly the influence of the harvest period, represented by blue curved lines (Figure 2b). The fourth factor observed was the growing season, where a slight tendency to group the samples could be seen. However, samples from two growing seasons studied exceptionally overlapped and could not be distinguished from each other, as shown in the biplot provided (Figure 2b). This suggests that the influence of the growing season was negligible. Nevertheless, this should be interpreted with caution, as it is possible that the weather conditions measured in the two seasons were not different enough to affect the VOOs phenolic compounds and OS and thus can be distinguished by PCA.

### 3.5. Molecular Markers for Traceability of Virgin Olive Oils from Oblica and Leccino Cultivars

The knowledge of the molecular profile of olive cultivars is the basis for verification of cultivars used for olive oil production and thus for successful traceability of VOOs. To support chemical analyses, DNA analysis can be used to verify the authenticity of olive oil because it is not affected by the environment and food processing, making it a good resource for comparing different genetic materials. In recent decades, molecular markers have become important in olive genotyping [28,73], population genetics [74] and product traceability [75,76].

DNA extraction from leaves and VOOs of Oblica and Leccino cultivars was successfully achieved (Table 6). DNA from leaves had optimal quality and a concentration of 100 ng/μL, whereas DNA extracted from VOOs had lower concentration, 4.3–21.8 ng/μL (respectively for the two cultivars). Values of 260/280 were in range from 1.66 to 2.12. Lower concentrations of extracted DNA from VOOs were also confirmed in some previous studies, reporting low yield but pure DNA isolation [29]. For the Leucocarpa cultivar, the concentration of DNA extracted from VOO was only 5.0 ng/μL, and was significantly lower than that obtained from leaves [77]. Since VOOs contain high amounts of polyphenols, polysaccharides, and proteins as some of the polymerase inhibitors [77,78]; this is also reflected in the lower values of the 260/230 ratio obtained in this study (Table 6).

Despite the DNA isolated from the VOOs was partially degraded, it was successfully amplified by five of the ten pairs of PCR primers tested. For the remaining five microsatellite loci, the isolated DNAs was not sufficiently pure for PCA amplification, most likely due to the presence of polymerase inhibitors [29,79]. Breton et al. [80] found that microsatellite loci were not amplified in 20% of VOO samples. The sizes of alleles obtained by amplification of DNA extracted from Oblica and Leccino samples are listed in Table 7.

Although great efforts will be needed by molecular laboratories and research groups to replicate and standardize the results and make this method routine for traceability assessment [29], SSR profiles of VOOs obtained from Oblica and Leccino cultivars were successfully amplified by microsatellites and correspond to the SSR profile of DNA isolated from the leaves of these cultivars, confirming the efficiency of SSR markers in the authentication of VOOs.

## 4. Conclusions

Results of data analysis of studied VOOs have shown that agro-climatic factors and time of olive harvest cause changes in VOO phenolic compound composition and oxidative stability, although varietal oils do not respond equally. Environmental conditions influenced the concentrations of the majority phenolic compounds, and the strongest negative correlation of precipitation in the ripening period with TOS concentrations was seen. By 20% higher TOS values were recorded in Leccino VOOs obtained from the colder and rainier growing season, whilst there were almost no differences in TOS among Oblica VOOs from two growing seasons. Pinoresinol proved to be a compound on which changes in rainfall and temperature did not influence. Regarding the growing site influence, generally, cultivars showed higher content of secoiridoid fraction and lower concentration of vanillic acid and pinoresinol in a colder and higher altitude growing site. Simple phenols were generally found to decrease with MI incensement. For the secoiridoids, monocultivar VOOs respond univocal; in Leccino VOOs were found to peak in the mid-harvest oils while in Oblica VOOs a constant decrease with maturation was observed. Oxidative stability was influenced by all studied factors and high positive correlations were observed with C18:1/C18:2 ratio and DMOdA concentration. Between the four main investigated factors, cultivar followed by geographical site exhibited the highest influence on the phenolic compound composition and OS of obtained VOOs revealed by the multivariate statistic. 

The knowledge of the molecular profile of VOOs is the basis of the authenticity that was presented for the first time for Croatian VOOs. Moreover, the rationale behind the current study was to better understand the causes of the differences in phenolic compound composition and oxidative stability, which is of great interest for VOOs producers for the targeted and timely harvest and processing and prediction of the shelf life of oils. 

## Figures and Tables

**Table 1:** Phenolic profile of Oblica virgin olive oils during ripening obtained from two distinct olive orchards (Kaštela and Šestanovac) in two successive growing seasons.

Factor			Phenols (mg kg^−1^)														
			HYT	TYR	VAC	VAN	PCM	FER	DMOdA	OAgl-A	OA-dA	DMLdA	LAgl-A	TOS	PIN	LUT	API
**2011**	**Kaštela**	**1.**	8.07 b	8.63 b	0.71 a	1.28 b	2.22 a	0.33 a	185.1 d	107.2 d	19.39 e	120.7 d	14.23 d	449.9 e	32.99 a	4.49 e	0.54 f
		**2.**	5.13 e	6.26 d	0.53 b	1.24 b	1.27 b	0.25 c	109.3 e	98.8 e	17.81 e	100.2 e	10.4 e	339.7 f	32.73 a	4.90 e	0.50 f
		**3.**	3.31 f	4.61 f	0.44 c	0.45 d	0.58 c	0.23 c	95.3 f	70.0 f	6.05 f	85.1 f	6.67 f	264.8 g	32.61 a	5.00 de	0.65 e
		**4.**	3.01 f	4.27 f	0.41 c	0.23 d	0.58 c	0.21 c	78.8 g	66.1 f	4.53 f	85.5 f	5.13 f	243.6 h	22.48 c	5.65 d	0.71 e
	**Šestanovac**	**1.**	11.07 a	10.31 a	0.38 d	1.69 a	0.58 c	0.28 b	277.2 a	197.1 a	84.74 a	163.5 a	45.9 a	768.4 a	19.88 d	7.85 c	0.88 d
		**2.**	10.79 a	8.43 bc	0.22 e	1.39 b	0.58 c	0.29 b	256.1 b	155.3 b	51.95 b	138.4 b	43.53 a	648.9 b	21.60 c	8.29 c	1.04 c
		**3.**	7.16 c	8.07 c	0.21 e	0.73 c	0.58 c	0.13 d	254.1 b	140.9 c	43.18 c	118.6 d	38.15 b	594.9 c	22.86 c	9.51 b	1.40 b
		**4.**	6.16 d	5.28 e	0.21 e	0.46 cd	0.58 c	0.04 e	213.4 c	134.6 c	33.59 d	125.7 c	34.35 c	542.6 d	26.17 b	12.87 a	1.92 a
**2012**	**Kaštela**	**1.**	2.69 e	3.91 f	0.37 b	0.47 a	2.06 a	0.36 c	108.1 d	96.6 d	8.2 d	124.3 d	12.2 e	351.1 d	17.67 d	3.47 d	0.71 de
		**2.**	2.71 e	4.70 ef	0.36 b	0.41 b	2.01 ab	0.42 b	91.5 e	87.8 e	8.1 d	98.7 e	13.6 e	301.1 e	26.27 b	3.65 d	0.72 de
		**3.**	2.22 e	6.17 cd	0.35 b	0.30 c	1.87 c	0.47 b	91.1 e	30.4 f	2.9 e	98.3 e	11.6 e	235.8 f	34.62 a	3.36 d	0.78 de
		**4.**	2.21 e	5.14 d	0.28 c	0.27 d	1.52 d	0.42 b	89.9 e	24.3 f	1.6 e	85.1 f	9.30 e	211.3 g	28.47 b	2.68 d	0.56 e
	**Šestanovac**	**1.**	9.78 b	10.87 a	0.49 a	0.41 b	1.90 bc	0.66 a	271.6 a	164.0 a	84.3 b	166.5 a	45.4 b	731.6 a	22.43 c	7.67 c	1.06 cd
		**2.**	10.84 a	9.04 b	0.46 a	0.29 c	1.51 d	0.70 a	234.8 b	187.0 b	99.7 a	137.1 b	51.8 a	715.9 a	22.78 c	8.23 bc	1.27 bc
		**3.**	8.54 c	6.72 c	0.48 a	0.30 c	0.96 e	0.68 a	233.0 b	153.4 c	86.5 b	130.6 c	37.0 c	646.2 b	17.10 d	9.51 b	1.55 ab
		**4.**	6.48 d	5.44 de	0.37 b	0.23 d	0.96 e	0.48 b	223.4 c	146.5 c	77.8 c	123.6 d	31.3 d	607.4 c	11.61 e	10.96 a	1.84 a
***GSn***	**2011**		6.84 a	6.98 a	0.39	0.93 a	0.87 b	0.22 b	183.6 a	121.2 a	32.65 b	117.2 b	24.8 b	481.6 a	26.40 a	7.32 a	0.96 b
	**2012**		5.68 b	6.50 b	0.4	0.34 b	1.60 a	0.52 a	167.8 b	111.2 b	46.09 a	120.4 a	26.4 a	475.0 b	22.62 b	6.19 b	1.06 a
	***F***		340.75	43.25	1.70	1192.458	8121.64	6974.75	875.4	198.0	1634.02	49.3	24.97	24.5	495.18	131.79	17.969
	***p***		***	***	ns	***	***	***	***	***	***	***	***	***	***	***	***
***GS***	**Kaštela**		3.67 b	5.46 b	0.43 a	0.58 b	1.51 a	0.34 b	106.1 b	72.6 b	8.54 b	99.7 b	10.37 b	299.6 b	28.48 a	4.15 b	0.65 b
	**Šestanovac**		8.85 a	8.02 a	0.35 b	0.69 a	0.96 b	0.41 a	245.4 a	159.8 a	70.21 a	137.9 a	40.91 a	657.0 a	20.55 b	9.36 a	1.37 a
	***F***		6848.33	1211.06	206.16	38.699	4766.76	394.52	68529.6	14903.9	34402.61	6953.7	8258.37	71634.4	2160.79	2803.60	868.894
	***p***		***	***	***	***	***	***	***	***	***	***	***	***	***	***	***
***HP***	**1.**		7.9 a	8.43 a	0.49 a	0.97 a	1.69 a	0.41 a	210.4 a	141.2 a	49.14 a	143.7 a	29.4 a	575.2 a	23.24 b	5.87 c	0.81 c
	**2.**		7.37 b	7.11 b	0.39 b	0.83 b	1.34 b	0.42 a	172.8 b	132.2 b	44.37 a	118.5 b	29.81 a	501.4 b	25.85 a	6.26 c	0.88 c
	**3.**		5.31 c	6.40 c	0.37 b	0.44 c	1.00 c	0.38 b	168.3 b	98.6 c	34.62 b	108.1 c	23.33 b	435.4 c	26.81 a	6.85 b	1.09 b
	**4.**		4.46 d	5.03 d	0.32 c	0.31 c	0.91 c	0.29 c	151.3 c	92.8 c	29.37 c	104.9 c	20.00 c	401.2 d	22.18 b	8.04 a	1.26 a
	***F***		684.16	371.76	172.44	330.704	1946.66	262.39	2194.8	1134.0	733.54	1469.3	202.73	3310.6	161.05	92.37	71.399
	***p***		***	***	***	***	***	***	***	***	***	***	***	***	***	***	***

Means marked by different lowercase letters (a–h) in column (for each growing season) and for each main factor (growing season, growing site, and harvest period) are significantly different (Tukey’s test, *p* ≤ 0.05). Significance: ***—*p* ≤ 0.001, ns—not significant. Identification of main factors; GSn—growing season; GS—growing site; HP—Harvest period (1–4). Identification of phenolic compounds: HYT—hydroxytyrosol; TYR—tyrosol; VAC—vanillic acid; VAN—vanillin; PCM—*p*-coumaric acid; FER—ferulic acid; DMOdA—dialdehyde form of decarboxymethyl oleuropein aglycone; OAgl-A—dialdehyde form of oleuropein aglycone; OA-dA—aldehyde form of oleuropein aglycone; DMLdA—dialdehyde form of decarboxymethyl ligstroside aglycone; LAgl-A—aldehyde form of ligstroside aglycone; TOS—total secoiridoid content; PIN—pinoresinol; LUT—luteolin; API—apigenin.

**Table 2:** Phenolic profile of Leccino virgin olive oils during ripening obtained from two distinct olive orchards (Kaštela and Šestanovac) in two successive growing seasons.

Factor			Phenols (mg kg^−1^)														
			HYT	TYR	VAC	VAN	PCM	FER	DMOdA	OAgl-A	OA-dA	DMLdA	LAgl-A	TOS	PIN	LUT	API
**2011**	**Kaštela**	**1.**	3.32 de	3.81 d	1.14 b	0.39 c	0.58 c	0.26 bc	81.0 d	25.1 d	1.16 c	32.9 h	6.09 e	148.3 g	5.39 e	1.90 g	0.34 e
		**2.**	3.82 d	1.25 e	1.28 a	0.44 c	0.58 c	0.17 bc	80.2 d	28.0 d	1.40 c	67.5 f	3.02 f	180.3 f	10.49 c	2.68 ef	0.58 e
		**3.**	3.03 ef	1.08 ef	0.78 c	0.26 c	0.58 c	0.11 d	83.2 d	29.3 d	1.23 c	83.9 e	2.19 g	200.1 e	11.94 b	3.48 cd	1.32 c
		**4.**	1.18 g	0.81 f	0.35 d	0.22 c	0.58 c	0.11 d	60.6 e	15.4 e	0.90 c	42.9 g	2.06 g	124.5 h	13.20 a	3.59 c	1.87 a
	**Šestanovac**	**1.**	8.90 a	11.39 b	0.30 d	1.18 ab	1.50 a	0.61 a	137.5 c	135.0 b	5.52 b	133.6 b	11.80 a	426.9 c	4.73 e	2.23 fg	0.91 d
		**2.**	5.71 b	12.80 a	0.29 d	1.41 a	1.28 b	0.50 a	186.5 a	168.6 a	5.36 b	142.7 a	10.96 b	517.8 a	6.41 d	4.18 b	1.45 bc
		**3.**	4.62 c	7.02 c	0.25 d	0.90 b	0.45 d	0.27 b	176.8 b	170.6 a	7.13 a	124.2 c	8.80 c	495.1 b	6.83 d	5.18 a	1.64 ab
		**4.**	2.48 f	5.48 d	0.12 e	0.88 b	0.28 e	0.13 cd	138.1 c	84.4 c	2.12 c	93.3 d	8.07 d	329.4 d	7.28 d	3.06 de	1.01 d
**2012**	**Kaštela**	**1.**	4.47 b	4.56 d	0.95 a	0.73 b	0.54 de	0.21 bc	102.6 h	29.2 e	1.66 d	91.0 b	5.79 f	231.2 g	10.92 ab	2.27 c	1.15 c
		**2.**	3.03 c	6.40 ab	0.81 b	0.74 b	0.36 e	0.18 cd	116.2 g	24.3 f	1.14 d	72.0 c	9.64 cd	224.9 g	10.83 ab	3.15 bc	1.44 d
		**3.**	2.80 cd	6.55 ab	0.63 c	0.67 b	1.25 b	0.14 de	183.9 e	20.3 g	1.85 d	66.1 d	9.46 d	283.1 e	11.43 a	4.35 a	1.85 a
		**4.**	2.33 d	4.78 d	0.43 d	0.27 c	0.73 cd	0.12 e	160.4 f	15.5 h	1.44 d	58.6 e	8.18 e	245.6 f	10.68 b	2.88 bc	1.55 b
	**Šestanovac**	**1.**	4.09 b	7.28 a	0.25 e	1.73 a	0.75 cd	0.27 a	276.3 d	40.1 d	6.50 a	73.4 c	12.31 a	409.7 d	6.39 c	3.44 ab	0.46 d
		**2.**	5.72 a	6.00 bc	0.23 ef	0.64 b	0.82 c	0.23 abc	347.9 a	90.6 a	5.46 ab	109.4 a	11.14 b	566.4 a	6.91 c	3.55 ab	0.59 d
		**3.**	5.50 a	5.29 cd	0.23 ef	0.61 b	1.67 a	0.24 ab	307.4 b	67.2 b	4.69 b	92.2 b	10.52 bc	483.9 b	6.46 c	2.55 bc	0.56 d
		**4.**	4.00 b	5.06 cd	0.21 f	0.62 b	0.81 c	0.18 cde	291.6 c	62.8 c	3.11 c	93.1 b	9.44 d	464.9 c	6.37 c	2.25 c	0.71 d
***GSn***	**2011**		4.13 a	5.46 b	0.56 a	0.71	0.73 b	0.27 a	117.9 b	82.0 a	3.1	90.1 a	6.63 b	302.8 b	8.28 b	3.29 a	1.14 a
	**2012**		3.9 b	5.74 a	0.47 b	0.75	0.87 a	0.20 b	223.2 a	43.6 b	3.23	81.9 b	9.56 a	363.7 a	8.75 a	3.05 b	1.04 b
	***F***		5.50	16.12	139.79	2.946	72.910	61.143	41232.8	7356.45	1.209	299.1	1465.47	8109.68	40.65	9.913	17.363
	***p***		***	**	***	ns	***	***	***	***	ns	***	***	***	***	***	***
***GS***	**Kaštela**		3.00 b	3.65 b	0.79 a	0.47 b	0.65 b	0.16 b	108.4 b	23.3 b	1.35 b	64.3 b	5.80 b	204.7 b	10.61 a	3.04 b	1.26 a
	**Šestanovac**		5.13 a	7.54 a	0.23 b	1.00 a	0.94 a	0.30 a	232.7 a	102.3 a	4.99 a	107.7 a	10.39 a	461.8 a	6.42 b	3.30 a	0.91 b
	***F***		1247.55	3036.17	4708.69	474.411	335.724	219.021	57410.5	31271.33	934.354	8455.3	3595.34	144401.88	3258.46	13.201	204.240
	***p***		***	***	***	***	***	***	***	***	***	***	***	***	***	***	***
***HP***	**1.**		5.19 a	6.76 a	0.66 a	1.01 a	0.84 b	0.34 a	149.3 c	57.3 c	3.71 a	82.7 c	9.02 a	304.0 c	6.86 c	2.46 d	0.71 b
	**2.**		4.57 b	6.61 a	0.65 b	0.81 b	0.76 c	0.27 b	182.6 a	77.8 a	3.34 a	97.8 a	8.69 b	372.4 a	8.66 b	3.39 b	1.01 c
	**3.**		3.99 c	4.99 b	0.47 c	0.61 c	0.99 a	0.19 c	187.7 a	71.7 b	3.73 a	91.5 b	7.74 c	365.5 b	9.17 a	3.89 a	1.34 a
	**4.**		2.50 d	4.03 c	0.28 d	0.50 c	0.60 c	0.13 c	162.6 b	44.4 d	1.89 b	71.9 d	6.94 d	291.1 d	9.38 a	2.95 c	1.28 a
	***F***		366.57	349.79	495.37	85.885	102.889	88.614	1187.4	1123.37	53.084	568.2	152.07	3792.02	244.49	68.907	141.453
	***p***		***	***	***	***	***	***	***	***	***	***	***	***	***	***	***

Means marked by different lowercase letters (a–h) in column (for each growing season) and for each main factor (growing season, growing site, and harvest period) are significantly different (Tukey’s test, *p* ≤ 0.05). Significance: ** *p* ≤ 0.01; ***—*p* ≤ 0.001, ns—not significant. Identification of main factors; GSn—growing season; GS—growing site; HP—Harvest period (1–4). Identification of phenolic compounds: HYT—hydroxytyrosol; TYR—tyrosol; VAC—vanillic acid; VAN—vanillin; PCM—*p*-coumaric acid; FER—ferulic acid; DMOdA—dialdehyde form of decarboxymethyl oleuropein aglycone; OAgl-A—dialdehyde form of oleuropein aglycone; OA-dA—aldehyde form of oleuropein aglycone; DMLdA—dialdehyde form of decarboxymethyl ligstroside aglycone; LAgl-A—aldehyde form of ligstroside aglycone; TOS—total secoiridoid content; PIN—pinoresinol; LUT—luteolin; API—apigenin.

**Table 3:** Correlation factors of phenolic composition of virgin olive oil and climatic parameters (rainfall and mean daily temperature; Table S1) in the period of intensive olive fruit growth and ripening.

Parameter	Rainfall					Mean Daily Temperature				
	Jul.	Aug.	Sep.	Oct.	Nov.	Jul.	Aug.	Sep.	Oct.	Nov.
Hydroxytyrosol	ns **	0.291	−0.459	−0.364	ns	−0.318	−0.579	ns	ns	ns
Tyrosol	ns	0.345	ns	ns	ns	ns	−0.467	ns	ns	ns
Vanillic acid	ns	−0.250	ns	−0.441	ns	ns	0.404	0.322	0.591	0.708
Vanillin	0.401	0.485	−0.352	−0.376	ns	−0.434	−0.478	ns	ns	−0.665
*p*-coumaric acid	−0.396	−0.363	0.353	ns	ns	0.402	0.332	−0.291	0.470	0.694
Ferulic acid	−0.279	ns	ns	ns	ns	ns	ns	−0.411	ns	ns
DMOdA *	ns	ns	−0.272	ns	0.652	ns	−0.461	−0.595	−0.328	ns
OAgl-A	0.337	0.467	−0.512	ns	ns	−0.444	−0.695	ns	−0.435	−0.632
OA-dA	ns	ns	−0.257	ns	0.511	ns	−0.347	−0.327	ns	ns
DMLdA	ns	0.340	−0.302	ns	ns	−0.264	−0.523	−0.254	−0.370	−0.501
LAgl-A	ns	ns	ns	ns	ns	ns	−0.422	−0.333	ns	ns
TOS	ns	ns	ns	−0.757	0.557	ns	−0.535	−0.595	−0.594	ns
Pinoresinol	ns	ns	ns	ns	ns	ns	ns	ns	ns	ns
Luteolin	ns	0.266	−0.354	ns	ns	−0.274	−0.455	ns	−0.427	ns
Apigenin	ns	ns	ns	0.345	ns	ns	ns	ns	−0.349	ns

* Identification: DMOdA-dialdehyde form of decarboxymethyl oleuropein aglycone; OAgl-A-dialdehyde form of oleuropein aglycone; OA-dA-aldehyde form of oleuropein aglycone; DMLdA-dialdehyde form of decarboxymethyl ligstroside aglycone; LAgl-A-aldehyde form of ligstroside aglycone; TOS—total secoiridoid content. ** Statistically significant difference at *p* ≤ 0.05; ns—correlation between parameters not significant *p* ≤ 0.05.

**Table 4:** Correlation factors of virgin olive oil oxidative stability and climatic parameters (rainfall and mean daily temperature; Table S1) in the period of intensive growth and ripening of olive fruits.

Parameter	Rainfall					Mean Daily Temperature				
	Jul.	Aug.	Sep.	Oct.	Nov.	Jul.	Aug.	Sep.	Oct.	Nov.
Oxidative stability	ns *	ns	−0.340	ns	ns	ns	−0.573	−0.497	−0.389	−0.394

* Statistically significant difference at *p* ≤ 0.05; ns—correlation between parameters not significant *p* ≤ 0.05.

**Table 5:** Correlation factors between phenolic compounds, tocopherols and fatty acids and oxidative stability of virgin olive oils.

Parameter	Oxidative Stability	Parameter	Oxidative Stability	Parameter	Oxidative Stability
HYT	0.569	Pinoresinol	−0.444	C18:3	−0.368
TYR	0.565	Luteolin	0.435	C20:0	ns
VAC	−0.372	Apigenin	0.409	C20:1	ns
VAN	0.303	TTC	0.310	C22:0	ns
PCA	ns	α-tocooherol	0.310	C24:0	−0.274
FER	0.252	γ-tocopherol	ns	SFA	ns
DMOdA	0.805	C 16:0	ns	PUFA	−0.601
OAgl-A	0.600	C 16:1	ns	MUFA	0.632
OA-dA	0.519	C18:0	ns	C 18:1/C 18:2	0.521
DMLdA	0.534	C 18:1	0.653	MUFA/SFA	0.428
LAgl-A	0.580	C18:2	−0.593	MUFA/PUFA	0.527

Statistically significant difference at *p* ≤ 0.05; ns—correlation between parameters not significant *p* ≤ 0.05. Identification of phenolic compounds: HYT-hydroxytyrosol; TYR-tyrosol; VAC—vanillic acid; VAN-vanillin; PCM-*p*-coumaric acid; FER-ferulic acid; DMOdA-dialdehyde form of decarboxymethyl oleuropein aglycone; OAgl-A-dialdehyde form of oleuropein aglycone; OA-dA-aldehyde form of oleuropein aglycone; DMLdA-dialdehyde form of decarboxymethyl ligstroside aglycone; LAgl-A-aldehyde form of ligstroside aglycone; TOS–total secoiridoid content; PIN-pinoresinol; LUT-luteolin; API–apigenin; TTC–total tocopherol content.

**Table 6:** Concentration and purity of isolated DNA from leaves of Oblica and Leccino cultivars and the corresponding monocultivar virgin olive oils.

Parameter	Oblica		Leccino	
	Leaf	VOO	Leaf	VOO
DNA concentration (ng/µL)	100	4.3, 17.3	100	5.7, 21.8
260/280	1.90	2.02, 1.66	1.92	2.12, 1.66
260/230	1.08	0.92, 0.71	1.41	1.02, 0.62

VOO—virgin olive oil.

**Table 7:** SSR microsatellite loci and allele profile of leaves and virgin olive oils of Oblica and Leccino cultivars.

SSR Locus	Allele Size (bp)		Allele Size (bp)	
	Oblica Leaf	Oblica VOO	Leccino Leaf	Leccino VOO
DCA03	243, 253	243, 253	243, 257	243, 257
DCA05	194, 202	194, 202	206, 208	206, 208
DCA09	162, 204	n.a.	162, 162	n.a.
DCA14	188, 188	n.a.	180, 180	n.a.
DCA17	103, 113	103, 113	113, 113	113, 113
DCA18	173, 175	173, 175	171, 175	171, 175
GAPU101	197, 199	197, 199	191, 219	191, 219
GAPU71B	120, 124	n.a.	120, 140	n.a.
GAPU103A	136, 174	n.a.	172, 184	n.a.
UDO43	176, 176	n.a.	210, 216	n.a.

n.a.—not amplified; VOO—virgin olive oil.

## Data Availability

The original contributions generated for this study are included in the article/Supplementary Material; further inquiries can be directed to the corresponding author.

## Supplementary Materials

The following supporting information can be downloaded at: https://www.mdpi.com/article/10.3390/antiox11030594/s1, Table S1: Climatic parameters, temperature (°C) and rainfall (mm), measured for two distinct growing sites (Kaštela and Šestanovac) in two successive growing seasons (2011 and 2012); Table S2: Fruit maturity index of Oblica and Leccino cultivars grown in Kaštela and Šestanovac.
