# Supplementary material for: Quantitatively Unraveling Hierarchy of Factors Impacting Virgin Olive Oil Phenolic Profile and Oxidative Stability
Source: Antioxidants (Basel). 2022 Mar 20;11(3):594. doi: 10.3390/antiox11030594 (PMC8945558; doi:10.3390/antiox11030594)
Supplement: Supplementary file 1 [file antioxidants-11-00594-s001.zip › antioxidants-1633838-supplementary.pdf]

antioxidants MDPI

SUPPLEMENTARY MATERIAL

Supplemental Table S1. Climatic parameters, temperature (°C) and rainfall (mm), measured for two distinct growing sites (Kaštela and Šestanovac) in two successive growing seasons (2011 and 2012).

Growing site - Kaštela								
	Temperature parameters (°C)						Rainfall (mm)	
	2011.			2012.			2011.	2012.
	Mean	Max	Min	Mean	Max	Min		
January	7.6	12.7	1.8	6.8	11.9	2.1	50.2	46
February	8.1	12.6	3.2	4.8	15.2	-1.6	33.4	38.2
March	10.6	14.9	3.6	12.2	16.8	7.9	97.6	0.8
April	15.9	20.6	9.5	13.6	20.5	6.6	9.6	117
May	19.6	26.1	14.5	18.5	24.2	13.2	89.4	48
June	24.5	28	20	24.7	30.1	18.8	34.4	35.6
July	25.1	31.3	20.7	28.2	31.4	21.8	130.8	8.8
August	26.6	31.5	22.9	27.4	33.4	23.4	1	0.2
September	24.2	28.3	18.8	21.7	25.2	16.2	62.8	157.4
October	16.4	23	10.1	17.1	22.6	7.8	193.2	160.2
November	12	17.8	8.7	15.1	18.8	12.1	103.6	63.8
December	10	15.5	4.2	8.1	15	2	78.2	265.4
**Growing site - Šestanovac**								
	Temperature parameters (°C)						Rainfall (mm)	
	2011.			2012.			2011.	2012.
	Mean	Max	Min	Mean	Max	Min		
January	5.8	11.8	0.1	4.6	9.7	-0.4	43.6	32.1
February	6.7	11.9	-0.5	1	11.8	-7.1	33.5	78.1
March	9	13.7	1.6	11.6	16.1	6.9	122.5	0
April	14.4	19	8	12.3	20.5	5.4	17.5	189.9
May	17.7	23.1	11.8	16.9	22.3	9.5	92.7	66.1
June	22.6	26.6	18.8	23.5	28.2	16.4	34.3	52.1
July	23.6	30.5	16.8	27.3	30.9	21.3	179.6	9
August	25.6	30.2	20.2	26.4	31.5	20.4	2.7	0
September	22.9	26.4	17.8	20.4	24.3	14.4	48.6	82.5
October	14.5	21.5	9.2	15.8	22.4	5.1	158.7	138.2
November	10.1	15.2	5.6	12.9	17.4	9	81.5	140.8
December	7.8	14.6	1.2	5.5	10.6	0	82	268

Supplemental Table S2. Fruit maturity index of Oblica and Leccino cultivars grown in Kaštela and Šestanovac

Growing season	Harvest period	Oblica		Leccino	
		Kaštela	Šestanovac	Kaštela	Šestanovac
2011	1.	0.00	0.00	2.49	1.41
	2.	0.48	0.44	3.50	2.01
	3.	1.70	2.94	4.00	3.96
	4.	3.94	3.87	4.05	4.10
2012	1.	0.37	0.29	2.11	1.05
	2.	0.79	0.56	2.31	1.72
	3.	2.12	1.27	3.15	2.56
	4.	2.86	2.84	3.48	3.37
